# On the origins and variation of nucleotide skews of archaeal genomes

**DOI:** 10.3389/fmicb.2026.1727296

**Published:** 2026-03-05

**Authors:** Adrien Paravel, Clémence Mottez, Romain Puech, Didier Flament, Hubert F. Becker, Hannu Myllykallio

**Affiliations:** 1 Laboratoire d’Optique et Biosciences (CNRS UMR7645, INSERM U1182), Ecole Polytechnique, Institut Polytechnique de Paris, Palaiseau, France; 2 Univ. Brest, Ifremer, UMR6197 BEEP, Plouzané, France; 3 Faculté des Sciences et Ingénierie, Sorbonne Université, Paris, France

**Keywords:** archaea, DNA replication and repair, non-canonical mismatch repair, nucleotide skews, replication origin

## Abstract

We have used nucleotide skews as the proxy to understand the evolution of archaeal genomes. Our genome-wide studies using substantial datasets suggest that translational selection and the nature of the genetic code are universally conserved determinants of asymmetric guanine and cytosine distributions. We propose that in the case of the majority of bacterial chromosomes, mutational processes and/or DNA repair also result in the strand-specific nucleotide skews. This is in stark contrast to what we observe for archaeal chromosomes and plasmids, and reveals that archaea have a greatly reduced ability to create mutations and/or repair DNA damage in a strand-specific manner. We suggest that in the future, the described computational and statistical approach will help to understand the evolutionary dynamics of the archaeal chromosomes through the tree of life.

## 1 Introduction

Archaea are a fascinating group of microorganisms with considerable evolutionary, environmental, and biotechnological interest that has been established since the pioneering work of Woese and Fox in the 1970s. From a molecular mechanistic point of view, studies on archaeal genomes have attracted extensive interest since the publication of the first archaeal genome sequence, which revealed that the architecture of archaeal circular chromosomes is very similar to that of bacteria in terms of gene density and operon structures. Nevertheless, archaeal replication proteins are more closely related to their eukaryotic counterparts than to their bacterial counterparts (Koonin et al., 2020). Strikingly, many archaeal DNA replication proteins, including DNA primase, replicative helicase, and DNA polymerase, are unrelated to their bacterial counterparts, raising questions about how functional parallels between semi-conservative and bidirectional DNA replication have evolved in the two prokaryotic domains. Moreover, despite the overall structure of archaeal replication origins being maintained during evolution (Gehring et al., 2017; Hawkins et al., 2013), these archaeal sequence elements necessary for the site-specific initiation of DNA replication are non-essential. Therefore, this demonstrates that it is possible to replicate an entire archaeal genome solely through recombination-dependent initiation (Hawkins et al., 2013; Hogrel et al., 2020; Hogrel et al., 2018). Indeed, in some archaea and cyanobacteria, the use of multiple replication origins and alternative replication mechanisms has been observed [for the recent review, see (Dulermo, 2025)]. Notably, stochastic cell-to-cell variation in haloarchaeal DNA replication and repair processes has been observed (Delpech et al., 2018), and the archaeal replication origin usage may be dependent on the growth phase (Mc Teer et al., 2024). In addition, recombination-associated DNA synthesis has been biochemically reconstituted using DNA polymerases (PolD and/or PolB) and the recombinase RadA, suggesting that interplay between origin-dependent and independent mechanisms can be used to initiate DNA replication in archaea (Mc Teer et al., 2024; Hawkins et al., 2013). Such observations raised the possibility that the mechanisms shaping the evolution of archaeal genome sequences may be distinct from those of the majority of bacteria.

As a basic reminder, Erwin Chargaff’s first parity rule states that, in double-stranded DNA, the molar ratios of guanine and cytosine as well as adenine and thymidine are identical, which indeed reflects base pairing in the DNA duplex. Later, he extended this observation to his second parity rule, indicating that these ratios also hold for individual strands of dsDNA and entire genomes (Rudner et al., 1968). The basis for this evolutionarily conserved phenomenon, except for mitochondria and ssDNA viruses, remains poorly understood. It is even argued that this DNA sequence symmetry has no biological basis but arises from randomness (Fariselli et al., 2021). However, at the whole-genome level, local deviations in nucleotide composition result in asymmetries in base composition. These are referred to as nucleotide “skews,” indicating, for instance, the local excess of guanine over cytosine (GC skew) that can be presented as (G − C)/(G + C) in a given genome window. These skews locally violate the second parity rule and have biological origins (Rocha et al., 2006). In particular, combinations of strand-specific biases in DNA replication and repair, transcription, regulation of gene expression, gene density and orientation, and translational codon biases contribute to local deviations from the second parity rule in bacterial genomes (Karlin, 1999; Lopez et al., 1999; Lobry, 1996). Causes for local deviations from Chargaff’s second parity rule can be broadly defined as translational selection and strand-specific mutational processes. Due to the redundancy of the genetic code and codon position-specific variation, the influence of translation, and direction of transcription of the protein-encoding genes, on the amplitude of the total genome-wide GC skew can be quantified by studying the GC skew in the first and the second codon positions (Saier, 2019). Gene Strand Bias (GSB) has been recently analyzed in bacteria (Atre et al., 2024; Tomasch et al., 2024) but remains relatively poorly characterized in archaea. On the other hand, mutation-related variation on the nucleotide skews is detectable on the third degenerate codon position or within non-coding DNA sequences. Notably, the linkage of the GC skew formation and DNA replication has been experimentally demonstrated in a bacterial model (Bhagwat et al., 2016; Kono et al., 2018). Moreover, strand-specific compositional asymmetries have been used to study possible DNA replication mechanisms in the double-stranded DNA viruses (Grigoriev, 1999).

## 2 Materials and methods

### 2.1 Datasets

Bacterial and archaeal genomic sequences and their annotations were obtained via NCBI GenBank. The GC and GSB skews, and their subcomponents, were retrieved from the SkewDB database [April 2025 version, (Hubert, 2022)]. This database provides the skew data used in this analysis, including 874 archaeal sequences (601 chromosomal and 273 plasmid sequences) and 52,963 bacterial sequences (43,682 chromosomal and 9,281 plasmid sequences). Further details are available in Supplementary Table S1.

### 2.2 Skew calculations

The cumulative GC skew curves were obtained by dividing each genomic sequence into windows of 4,096 bases. The following formula was applied to each window, assigning it a value between −1 and 1: [(G − C)/(G + C)], where G and C are the numbers of guanine and cytosine observed in the window, respectively. The curves were then plotted by adding the value of each window to the sum of the values of the previous windows. In contrast to the total GC skew, which has just been explained in the previous lines, the different GC skews (“sub-skews”) used in this study are obtained according to coding and non-coding regions. For GC skew calculated solely based on coding regions, the codons of each gene are extracted, and the nucleotides are separated according to their positions (1st, 2nd, or 3rd). The GC skew formula is then applied, considering a single fixed position within each codon. The non-coding GC skew is calculated by applying this same formula to non-coding regions only.

GSB curves were also calculated using a cumulative approach along the genomes. A counter was used to track the contribution of each nucleotide based on whether it belonged to a gene located on the positive strand or the complementary strand. This counter is incremented by 1 for each nucleotide belonging to a gene on the positive strand, or decremented by 1 for each nucleotide belonging to a gene on the complementary strand, and remains unchanged for nucleotides in non-genic regions. The GSB at a given position i corresponds to the cumulative sum of these contributions up to that position, thus reflecting the relative excess of genes on one or the other strand. This measurement makes it possible to identify genomic regions where genes are preferentially located on one strand. The GSB was plotted along the genome to visualize local and global variations in strand bias, complementing GC skew analyses.

Where indicated, it was necessary to offset some graphs to increase the visibility of the different figures. This was done by adding or subtracting the arbitrary values from the actual values to prevent the crossing over of the different curves. Consequently, the shapes and min/max, but not the amplitudes, of the different curves can be directly compared.

### 2.3 Comparative analyses and statistics

To study the distribution of guanine (G) and cytosine (C) nucleotides in archaeal genomes, we retrieved the genomic sequences of the 874 archaea, whose metadata are listed in the SkewDB database via NCBI GenBank. For each genome, the total number of G and C nucleotides was determined by scanning the FASTA sequences and counting the occurrences of each nucleotide. The visualization of the results represents one genome at each point, with the number of Gs on the *x*-axis and the number of Cs on the *y*-axis.

The Skew Index Test (skewIT) (Lu and Salzberg, 2020) calculates a single value representing the degree of GC imbalance for a genome. This method has been adapted and automated for deployment across all genomes analyzed in the SkewDB database. Due to the recommended selection criteria for its application (i.e., sequences annotated as complete and longer than 500,000 bases), only 43,825 raw genomic sequences from the 53,837 organisms analyzed were used. This explains why not all archaeal genomes were analyzed using the skewIT index.

We also estimated the fraction of the chromosome replicated as the leading strand, which has been denoted as “div” in the SkewDB database. The “div” value was calculated as the predicted proportion of the leading strand length relative to the total genome length. This value was obtained by the fitting of the CGC data [for details of the fitting procedure, see the reference (Hubert, 2022)].

The statistical tests used to compare the different distributions have been described in the figure legends and/or main text. All statistical analyses were performed using GraphPad Prism 10 [version 10.6.1 (799)] and/or Python 3.12.2.

### 2.4 Genome alignments

**Figure 1:**
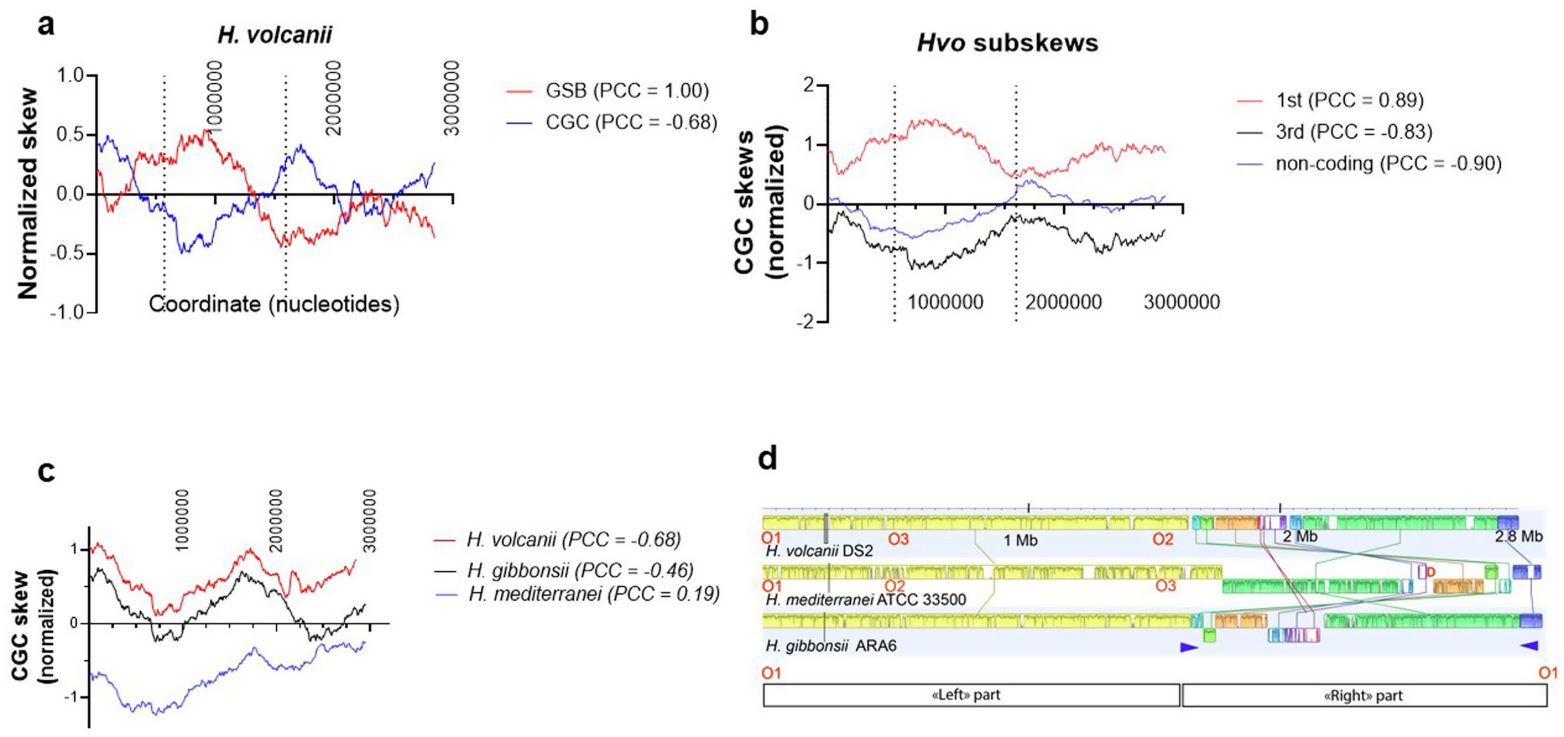
Examples of GSB and CGC skews for *Haloferax* spp. genomes. **(a)** Anticorrelation of *H. volcanii* DS2 GSB and CGC skews (PCC = −0.68). **(b)** The different CGC “subskews” calculated for the first and third codon positions as well as for the non-coding part of the genome. The figure illustrates that the first codon position correlates strongly with the GSB, whereas the two additional skews show anticorrelation. In panels **(a)** and **(b)**, the dotted vertical lines indicate the position of the replication origins found in three species. **(c)** Comparison of the CGC skews of three *Haloferax* species. **(d)** Progressive Mauve alignment of three *Haloferax* genomes. The alignment was performed using Geneious Prime® 2025.2.2 Build 2025-08-20 12:11 with the default settings. Color coding indicates the conserved synteny blocks. O1, O2, and O3 refer to the conserved replication origins of three analyzed genomes. D indicates the approximate position of the dormant replication origin that has only been identified in *H. mediterranei*. The blue triangles indicate the position of the two copies of the 16S-23S rRNA operon in three genomes.

The genome alignments of *Haloferax* spp. (Figure 1d) were performed using Progressive Mauve (implemented in Geneious Prime® 2025.2.2 Build 2025-08-20 12:11), using default parameters to identify conserved synteny blocks that were automatically colored according to their syntenic conservation.

## 3 Results

### 3.1 Nucleotide skews as a proxy for understanding the evolution of archaeal genomes

**Figure 2:**
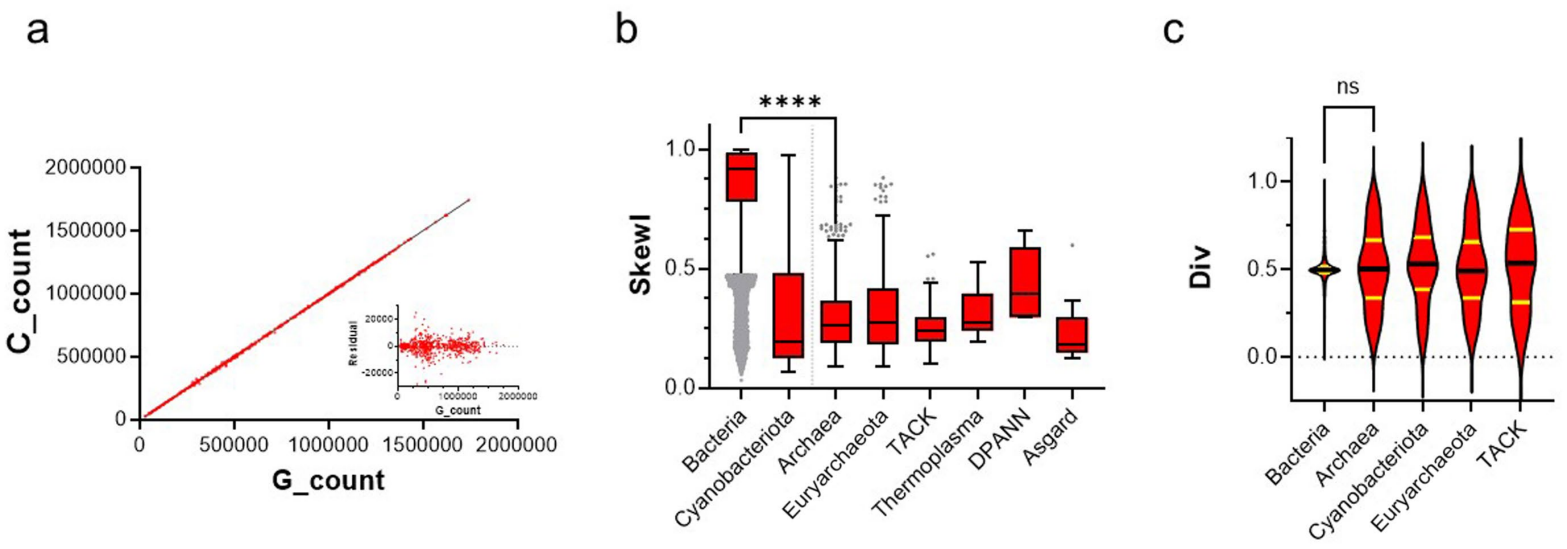
Statistical analyses of archaeal chromosomes. During this work, all statistical tests were performed using the GraphPad Prism 10 [version 10.6.1 (892)]. **(a)** Correlation between guanine and cytosine counts in all archaeal replicons of the dataset (874 sequences) with a linear (Pearson) correlation coefficient (*r*^2^) of 1.00. The inset indicates the residuals of the linear fit of the data, revealing only minor deviation from the observed linear correlation. **(b)** Distribution of skew index (SkewI) values for different taxonomic levels calculated using the sequences that passed the quality control. The distributions and whiskers were plotted using the Tukey method. Grey dots indicate the out-layer values. SkewI values between bacteria and archaea are statistically highly different (Kruskal-Wallis test, **** refers to the adjusted *p*-value < 0.0001). **(c)** Distribution of div values for different taxonomic levels; mean values are indicated with the black line, whereas quartile limits are indicated in yellow.

Here, we have combined quantitative nucleotide and GSB skews, together with statistical analyses, of complete archaeal and bacterial genome sequences to understand the diversification of molecular mechanisms shaping the archaeal genomes. As far as we are aware, this reasoning and our bioinformatics pipeline have not been previously used on the large datasets of archaeal genomes. This approach is further justified by earlier work revealing that the strength (amplitude) of the GC skew in most eubacteria is much higher than in archaea (Arakawa et al., 2009). However, the underlying molecular mechanisms remain poorly understood. We have investigated the origins of archaeal GC skew in complete archaeal reference genomes. We first confirmed a strong linear correlation (*r*^2^ = 1.00) between cytosine and guanine counts for single strands of available archaeal replicons of our extended dataset (Figure 2a), thus confirming that nucleotide counts of C and G nucleotides are (approximately) the same within a single strand of archaeal chromosomes. The obtained fit was of high quality for the vast majority of archaeal genomes, as the individual data points were well centered around the linear regression line (Figure 2a, inset). This conclusion regarding the symmetries of archaeal DNA sequences is consistent with an earlier analysis that used a less exhaustive dataset of 170 archaeal sequences (Fariselli et al., 2021). We next implemented the Skew Index (SkewI) test for archaeal genomes (for compilation of our earlier results with a smaller dataset, see (Mottez et al., 2023) and 10.5281/zenodo.8126182). This test was originally used for large-scale analyses of bacterial genomes (Lu and Salzberg, 2020). SkewI represents a single numerical value, ranging from 0 to 1, which indicates the degree of GC skewness of the complete archaeal genomes. Figure 2b and the Supplementary Figure S1 indicate that complete archaeal reference genomes have SkewI values with a mean value of 0.303 ± 0.16 (*n* = 632). However, this value is statistically significantly lower (*p*-value < 0.0001, Mann–Whitney test, two-tailed) than that of bacterial genomes (0.830 ± 0.22, *n* = 43,112). We did not observe a major variation in the SkewI between the different archaeal superphyla (one-way ANOVA test, corrected *p*-values > 1.0). Interestingly, cyanobacteria (cyanobacteriota) behaved in these analyses very similarly to archaeal species (0.24 ± 0.22, *n* = 278), and their SkewI was significantly lower (*p*-value < 0.001) than that of the other bacteria. We found that approximately 10% of archaeal genomes had a SkewI value higher than 0.5. This included examples from the TACK (*Thaumarchaeota*, *Aigarchaeota*, *Crenarchaeota*, and *Korarchaeota*) and Euryarchaeota superphyla, which presented the majority of the archaeal data points (TACK, *n* = 129; Euryarchaeota, *n* = 466).

Cumulative GC skews (CGC) refer to the sum of (G − C)/(G + C) in adjacent genome windows and are widely used to investigate changes in genome-wide nucleotide skews. We further quantified CGC skews in the archaeal genomes. This has recently been facilitated by the establishment of the Skew Database (SkewDB), which includes precalculated CGC skews for more than 50,000 bacterial and archaeal chromosomes and plasmids larger than 100 kb (Hubert, 2022). The use of cumulative nucleotide skews was originally developed to analyze bacterial and archaeal genome sequences (Grigoriev, 1998). The reported skews were calculated in successive windows of 4,096 nucleotides. SkewDB data agrees very well with our archaeal SkewI analyses (Figure 2b), indicating that the amplitude of bacterial CGC skews is, on average, ≈ 3.4 times higher than in archaea. We observe that bacterial values (except, e.g., for some cyanobacteria) are tightly centered around the value of 0.5001 ± 0.11 (Figure 2c), which indicates that replicons with equal or similar size initiate from a single well-defined replication origin and terminate at a well-defined terminus. We also plotted the predicted “div” values for the archaeal and cyanobacterial chromosomes and found that approximately 30% of archaeal div values are in the range of 0.4–0.6, which is the typical range of bacterial div values. Although the mean values for the different bacterial and archaeal div datasets were not significantly different (Figure 2c), their coefficients of variation (CV), or the ratio of the standard deviation to the mean, were much higher for archaea and cyanobacteriota than for the majority of bacteria. This indicates the marked variation of archaeal div values when compared to bacteria.

### 3.2 What are the causes of variable archaeal CGC skews?

**Figure 3:**
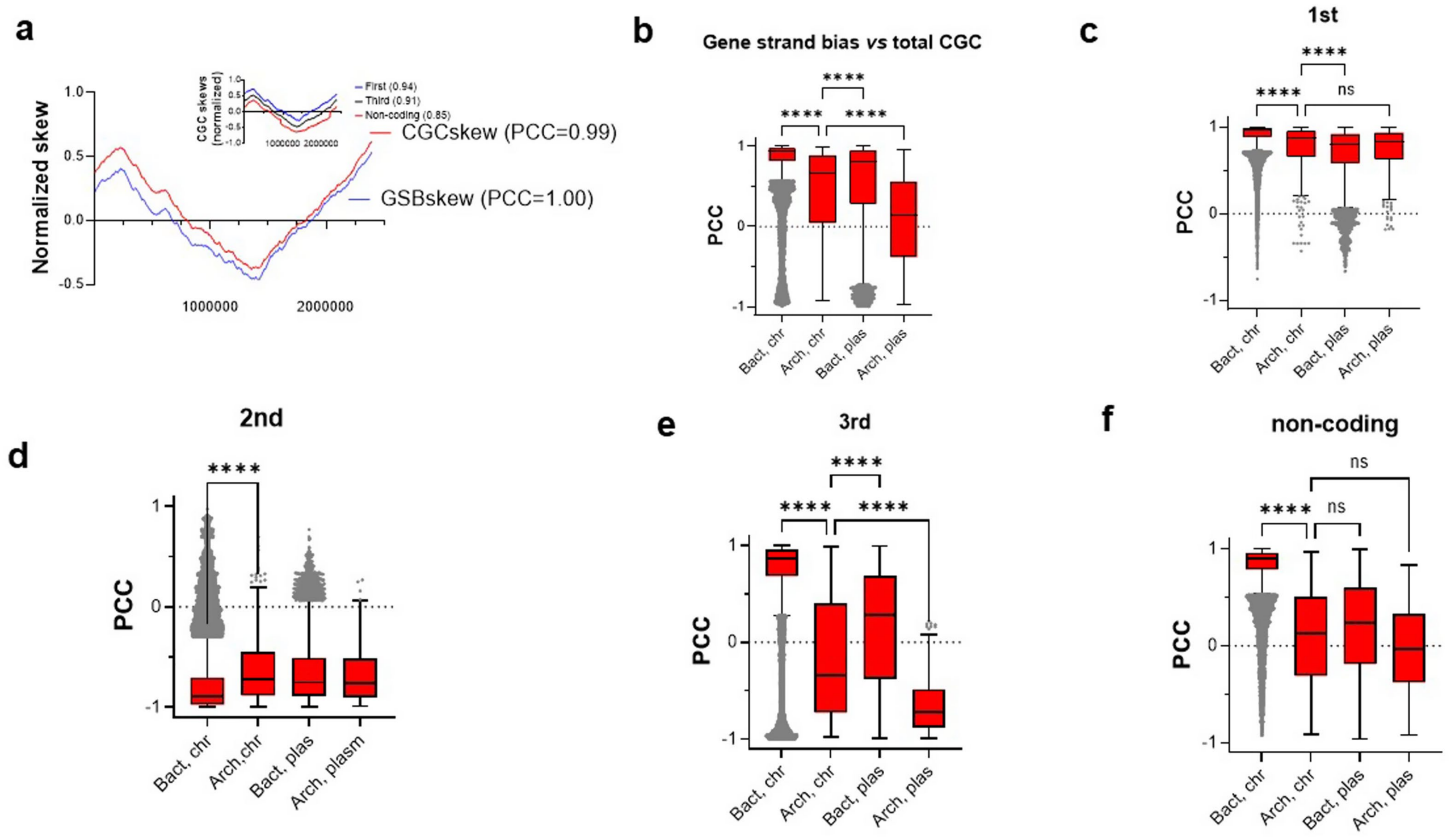
Correlation of gene strand bias (GSB) with cumulative GC (CGC) skews of prokaryotic chromosomes and plasmids. **(a)** CGC and GSB skews calculated over the entire genomic sequence NZ_AP019779.1 *Methanobrevibacter arboriphilus* strain SA chromosome. The graphs were normalized and offset to facilitate the simultaneous comparison of the different curves. The PCC values refer to the level of correlation with respect to the total GSB skew. GSBskew (PCC = 1.00) therefore refers to perfect self-correlation of GSBskew with itself. The inset indicates the CGCG skews in the first and third codon positions as well as in the non-coding DNA. The PCC with respect to GSB is indicated in parentheses. **(b)** Correlation of GSB with total CGC for the different types of replicons. Bact chr, bacterial chromosome; Arch chr, archaeal chromosome; Bact plas, bacterial plasmid; Arch plas; archaeal plasmid. Panels **(c–f)** show similar analyses for the different codon positions or non-coding DNA. **** refers to the adjusted *p*-value < 0.0001 with respect to archaeal chromosomes, whereas ns is an abbreviation for non-significant. In all panels, the outlier values are indicated by grey dots.

Our visual inspection of archaeal CGC and cumulative local gene strand biases identified many cases where the overall form and direction, but not the amplitude, of two curves were very similar (for example, for *M. arboriphilus*, see Figure 3a). It is also noteworthy that in this case, the shape of the CGC skews in the first and third codon positions as well as in non-coding sequences were highly similar (Figure 3a, inset). To test the correlation between these four parameters systematically, we determined the Pearson correlation coefficient (PCC) between the *local* gene strand biases (GSB) and the different CGC skews (total, codon specific, or non-coding DNA) in more than 50,000 prokaryotic genomes presented in the SkewDB (Figure 3b). These analyses revealed that in the case of bacterial chromosomes, GSB and the total CGC skews show very strong general correlation with a mean PCC of 0.81 ± 0.36 (Table 1; Figure 3b). The correlation between GSB and CGC for the archaeal chromosomes of 0.43 ± 0.54 indicates a more moderate correlation, which is in a similar range to that observed for bacterial plasmids (Figure 3). We also note that in archaeal plasmids, gene strand bias and CGC skews are, on average, not correlated. The important point is that the observed CVs are again very different (Table 1), thus indicating a marked variability of the different datasets in comparison to bacterial chromosomes. For additional comparison, we also indicated the percentage of genomes with the PCC higher than 0.5 between the local gene strand bias and CGC skews for the different classes of DNA sequences (Table 1, sixth column).

**Table 1:** The correlation of the local gene strand bias and total CGC skews in prokaryotic genomes and plasmids.

Class	Mean PCC	Standard deviation (SD)	Coefficient of variation (CV) %	Number of data points	PCC > 0.5
Bacterial chromosome (Bact, chr)	0.81	0.36	44.10	43,682	89.6
Archaea chromosome (Arch, chr)	0.43	0.54	126.4	601	59.9
Bacterial plasmid (Bact, plas)	0.54	0.56	104.3	9,281	66.3
Archaeal plasmid (Arch, plas)	0.08	0.36	661.9	273	26.9

To dissect the possible differential effects of translational selection and mutational effects on the archaeal CGC skews, we performed additional Pearson correlation analyses (Table 2; Figures 3c–f). These analyses revealed the highly significant positive correlation between the GSB and the first codon position, which was observed for bacterial and archaeal chromosomes and plasmids. In this analysis, a very high correlation was also observed between the GSB and both the 3rd codon position and non-coding DNA for bacterial chromosomes, but not for the other datasets. The observed strong negative correlation between the GSB and the second codon position CGC skew will be discussed below.

**Table 2:** The Pearson correlation (PCC) of the local gene strand bias and the different components of the CGC skews.

Class	1st position (mean PCC±SD)	2nd position (mean PCC±SD)	3rd position (mean PCC±SD)	Non-coding (mean PCC±SD)
Bact, chr	0.90 ± 0.16	−0.76 ± 0.33	0.67 ± 0.51	0.81 ± 0.26
Arch, chr	0.76 ± 0.27	−0.62 ± 0.33	−0.17 ± 0.61	0.09 ± 0.49
Bact, plas	0.70 ± 0.29	−0.65 ± 0.32	0.15 ± 0.59	0.20 ± 0.48
Arch, plas	0.72 ± 0.28	−0.67 ± 0.28	−0.64 ± 0.30	−0.03 ± 0.44

To investigate the evolutionary forces influencing the multi-origin halophilic archaeal main chromosomes (Hawkins et al., 2013; Norais et al., 2007; Yang et al., 2015), Figure 1 illustrates nucleotide skews for three diverse *Haloferax* genomes. Interestingly, *H. volcanii* GSB and CGC skews (Figure 1a) correlate negatively (PCC = −0.68), which is drastically different from the majority of the other archaea (Figure 3b). To better understand this curious phenomenon, we realized that *H. volcanii* CGC in the first codon position showed expected positive correlation with the local GSB (PCC = 0.89, see Table 2, second column for comparison). However, CGC skews at the third codon position, and non-coding DNA show the opposite direction from the GSB skew with the PCC values −0.83 and −0.90, respectively (Figures 1a,b, see Table 2, columns four and five for comparison). Figure 1c illustrates that, like *H. volcanii*, the *H. gibbonsii* (PCC = −0.46) and *H. mediterranei* (PCC = 0.19) genomes do not demonstrate the typical strong positive correlation between the CGC and GSB skews observed for the prokaryotic chromosomes (for comparison, see Figures 3a,b). However, the overall shape of the CGC skews is conserved in three *Haloferax* genomes.

We also constructed a whole-genome alignment between these *Haloferax* species using Mauve (Darling et al., 2004). Mauve allows constructing multiple genome alignments and is capable of detecting large-scale evolutionary events such as rearrangement and inversion in prokaryotic genomes. In these presentations, genomes are represented as horizontal lines, whereas homologous segments are represented as colored and connected blocks across three genomes. This analysis revealed that the analyzed *Haloferax* genomes can be divided into two major parts. The first part corresponds to the left part in the linear genome representation (Figure 1d), ranging from 0 to ≈ 1.6 Megabases, which shows a high level of synteny between the three genomes. The second genome portion corresponds to the region approximately ranging from 1.6 to 2.8 Megabases with many large-scale evolutionary events, including rearrangements and inversions. This portion also carries the dormant origin of *H. mediterranei* (Yang et al., 2015). Interestingly, these two genome portions are separated by the experimentally mapped replication origin in *H. volcanii* (O3, see Figure 1d) and *H. mediterranei* (02). This replication origin also coincides with the inversion point in the different *H. volcanii* subskews presented in Figure 1b (the replication origins are represented by the pointed vertical lines). Note that in this figure, the original numbering of the indicated replication origins was used.

## 4 Discussion

In this work, we have used genome-wide nucleotide skews as the proxy for studying the diversity of archaeal evolutionary dynamics.

We first confirmed that Chargaff’s second parity rule holds for archaeal chromosomes and plasmids used in our analyses (Figure 2a). Our statistical analyses indicated that SkewI, the quantitative measure of GC skewness, of archaeal genomes is three to four times smaller than has been previously observed for Bacteria (Figure 2b). We are aware that the SkewI method does not work well with incomplete or misassembled sequences, thus explaining why we have focused in this study on the archaeal reference genome sequences. In agreement with our analyses, SkewDB indicates an average of 7–8 excess Gs in 1000 base windows for archaea, which is much lower than what is observed for bacteria (23–25 excess G nucleotides). A priori, this could be potentially linked to the lower rate of spontaneous mutations or unusual mutational patterns in polyploid archaea (Mackwan et al., 2007; Soppa, 2022). However, as the ploidy number varies drastically among archaea (Soppa, 2022), we do not believe that a high ploidy number alone could explain the decreased amplitude of the archaeal GC skew.

Our results also clearly indicate that the vast majority of bacterial chromosomes have two equally sized replicons, as exemplified by the div values strongly centered around 0.5 (Figure 2c). We also found that approximately 30% of archaeal chromosomes have the predicted div values ranging from 0.4 to 0.6, suggesting that a single bidirectional replication origin is frequently used to replicate archaeal genomes. *Methanobrevibacter arboriphilis* strain SA analyzed in Figure 3a, is a representative of an archaeal chromosome with SkewI and div values of 0.79 and 0.51, respectively. This proposed “bacterial-like” replication mechanism of archaeal chromosomes has also been observed in experiments, for example, with the anaerobic hyperthermophile *Pyrococcus abyssi* (Myllykallio et al., 2000). The marked variance of archaeal and cyanobacteriota *div* values could simply reflect that the fitting procedure to determine the div values was developed for bacterial genomes with a high GC skewness. Therefore, the low amplitude of the archaeal CGC skews might complicate the fitting procedure, thus potentially missing archaeal genomes with a single replication origin. It is also feasible that the “low” and “high” div values are not simply artefacts. For instance, it is well established that many aforementioned archaea use multiple replication origins, which would decrease the predicted replicon size with div values smaller than 0.5. Moreover, the alternative origin-independent replication mechanisms have been observed both in archaea and cyanobacteria (Ohbayashi et al., 2020), also potentially contributing to the variation in the predicted div value. It is also noteworthy that the rolling circle replication mechanism, using only one strand of DNA duplex as template, has been proposed to result in the continuous GC skew without any shifts (Arakawa et al., 2009), which would be compatible with a div value of one. Consequently, we believe that the marked variation of archaeal div values has a biological meaning worth pursuing.

We observed that prokaryotic GSB and CGC skews very frequently have a highly similar shape (e.g., Figure 3a, PCC = 0.99 between two curves), particularly after normalization to allow easier visual comparison of the graphs. This suggests that gene orientation and/or translation strongly contribute to the formation of CGC skews. We hypothesized that the differential contributions of translational selection and mutational effects on the amplitude of CGC skew could explain the observed differences between bacteria and archaea. Figure 3a (inset) shows that the shape and directionality of the *M. arboriphilis* CGC skew in first and third codon positions, as well as non-coding DNA, are very similar. Moreover, the local maxima and minima of all the skews shown in the main figure or the inset of Figure 3a are perfectly aligned. We detected the strong correlation between GSB and the excess of G over C in the first codon position in bacteria and archaea. In contrast, the strong anti-correlation was observed for the second codon position. These observations are consistent with the current understanding of the genetic code and amino acid constraints (Saier, 2019). Each codon position influences specifically the nature of the amino acid to be incorporated into the polypeptide chain. Indeed, guanosine at the first codon position is a preferred nucleotide, whereas U/T and A at the second position are preferentially used to encode hydrophobic and hydrophilic amino acids, respectively. Therefore, local variation in GSB on the leading and lagging strands contributes to the GC skews of bacteria and archaea. Moreover, the observed bacterial correlation between GSB and G excess at the third codon position, as well as non-coding DNA, is readily detectable, indicating that mutational processes contributing to CGC are also strand-specific. It is of note that strand-biased cytosine deamination at the bacterial replication fork has been linked to the formation of GC skew (Kono et al., 2018), which has been experimentally demonstrated using accelerated laboratory evolution experiments using cytosine deaminase as a strand-specific DNA mutator. The GC skew phenomenon can therefore be used to determine the transition between the leading and lagging strands that correspond to the replication origins and termini. Common bacterial DNA repair mechanisms, such as the MutSL-dependent mismatch repair (MMR) and transcription-coupled nucleotide excision repair, also function in a strand-specific manner. Moreover, at least in some archaea, genome-wide nucleotide-resolution maps of DNA-embedded ribonucleotides reveal *oriC*-centered strand-switching profiles, also linking the replication origins with DNA repair processes (Moalic et al., 2025). Based on our observations (Table 2; Figure 3), we nevertheless suggest that the role of mutations influencing the extent of the archaeal GC skewness is less evident than for bacteria, likely reflecting the differences in the underpinning molecular events. Notably, in Archaea, the Okazaki fragments are much shorter than in Bacteria (Matsunaga et al., 2003). This could disfavor the formation of ssDNA at the replication fork, thus decreasing strand-specific cytosine deamination even though the replication speed in archaea is somewhat lower than in the model bacteria (O’Donnell et al., 2013). Moreover, in many archaea, MMR is dependent on the non-canonical mismatch-specific endonuclease NucS/EndoMS, thought to repair the mismatches by creating the double-strand break (Ren et al., 2009; Nakae et al., 2016; Ishino et al., 2018). A priori, this repair mechanism cannot function in a strand-specific manner. In addition, the rates of DNA repair of transcribed and non-transcribed strands in archaea are similar (Dorazi et al., 2007), which reduces the formation of transcription-dependent formation of the GC skew. We nevertheless stress that RNA polymerases also can cause strand-specific mutations on the non-template DNA strand during transcription (Grigoriev, 1999; Beletskii and Bhagwat, 1996; Grigoriev, 2004). Moreover, the MutSL-dependent MMR and transcription-coupled DNA repair mechanisms are not widespread in archaea (White and Allers, 2018). We hypothesize that the combination of these mechanistic differences can explain, at the molecular level, why the extent of the archaeal GC skew is limited when compared with bacteria. In addition, the stochastic and/or growth phase-dependent firing of archaeal replication origins may decrease the amplitude of the GC skew in archaea.

The whole-genome alignment between three diverse *Haloferax* species indicated that these genomes can be divided into two major parts. Whereas the first part (“left” part, Figure 1d) is highly syntenic between three genomes, the “right part” has undergone many high-level evolutionary changes. These two genome portions are separated by the experimentally mapped replication origin. The “right part” is also flanked by the two copies of the 16S-23S rRNA operon, thus facilitating their transcription from the leading strand. The total CGC of *H. volcanii* is difficult to rationalize in terms of the replication origin usage, as total GSB and CGC skews anticorrelated (PCC = −0.68). Interestingly, the lack of this correlation for archaeal genomes appears more common than previously assumed, as suggested by Figure 3b. However, the first codon position CGC correlates well with the GSB in this halophile (PCC = 0.89, Figure 1b). Consequently, *H. volcanii* Ori3 (03, Figures 1b,d) is expectedly located in the local *minima* in the first codon position CGC. However, unlike bacteria, this replication origin coincides with the local *maxima* of the third codon position and non-coding DNA CGC skews. This observation is very different from bacteria and further supports the notion that the mutational forces shaping the archaeal main chromosomes are very different from those operating on the majority of bacterial chromosomes. Similar deviations from canonical bacterial genome organizations have recently also been found less frequently in bacteria (see, e.g., (Kopejtka et al., 2019), therefore suggesting that complex and variable interactions between the different molecular mechanisms shape prokaryotic genome evolution. Strikingly, we found that in this respect archaeal chromosomes behave similarly to bacterial and archaeal plasmids (Figures 3e,f), further suggesting diminished capacity of the strand-specific DNA repair processes of archaeal chromosomes and prokaryotic plasmids. We also noticed that the dormant replication origin of *H. mediterranei* (indicated by the letter D in Figure 1d) is not conserved in the two other *Haloferax* species analyzed. This is an example of how evolutionary dynamics and the capture of extrachromosomal elements influence the replication origin and replicon evolution (Robinson and Bell, 2007; Wu et al., 2014).

In conclusion, we have used computational approaches and databases to analyze more than 50,000 prokaryotic replicons to dissect the level and variation of the archaeal GC skew. We present a new and unbiased interpretation of the archaeal nucleotide skews using the latest set of archaeal reference genomes available. Our results already indicate that translational selection is a universally conserved mechanism that shapes the evolution of bacterial and archaeal chromosomes and their plasmids. Strikingly, our analyses also revealed that the mutational spectra influencing the GC skewness of the archaeal genomes are different from those of bacteria, thus providing novel insight into evolutionary forces and molecular mechanisms shaping prokaryotic replicons. We also note that the replication timing and three-dimensional chromosomal organization influence the evolution of archaeal genomes (Flynn et al., 2010; Badel et al., 2022). In the future, this study will pave the way towards understanding the evolution of chromosomes through the archaeal tree of life.

## Data Availability

The raw data supporting the conclusions of this article will be made available by the authors, without undue reservation.

## References

[ref1] Arakawa K. Suzuki H. Tomita M. (2009). Quantitative analysis of replication-related mutation and selection pressures in bacterial chromosomes and plasmids using generalised GC skew index. BMC Genomics 10:640. doi: 10.1186/1471-2164-10-640, 20042086 PMC2804667

[ref2] Atre M. Joshi B. Babu J. Sawant S. Sharma S. Sankar T. S. (2024). Origin, evolution, and maintenance of gene-strand bias in bacteria. Nucleic Acids Res. 52, 3493–3509. doi: 10.1093/nar/gkae155, 38442257 PMC11040001

[ref3] Badel C. Samson R. Y. Bell S. D. (2022). Chromosome organization affects genome evolution in Sulfolobus archaea. Nat. Microbiol. 7, 820–830. doi: 10.1038/s41564-022-01127-7, 35618771 PMC9597579

[ref4] Beletskii A. Bhagwat A. S. (1996). Transcription-induced mutations: increase in C to T mutations in the nontranscribed strand during transcription in *Escherichia coli*. Proc. Natl. Acad. Sci. USA 93, 13919–13924. doi: 10.1073/pnas.93.24.13919, 8943036 PMC19468

[ref5] Bhagwat A. S. Hao W. Townes J. P. Lee H. Tang H. Foster P. L. (2016). Strand-biased cytosine deamination at the replication fork causes cytosine to thymine mutations in *Escherichia coli*. Proc. Natl. Acad. Sci. USA 113, 2176–2181. doi: 10.1073/pnas.1522325113, 26839411 PMC4776466

[ref6] Darling A. C. Mau B. Blattner F. R. Darling A. C. E. Perna N. T. (2004). Mauve: multiple alignment of conserved genomic sequence with rearrangements. Genome Res. 14, 1394–1403. doi: 10.1101/gr.2289704, 15231754 PMC442156

[ref7] Delpech F. Collien Y. Mahou P. Beaurepaire E. Myllykallio H. Lestini R. (2018). Snapshots of archaeal DNA replication and repair in living cells using super-resolution imaging. Nucleic Acids Res. 46, 10757–10770. doi: 10.1093/nar/gky829, 30212908 PMC6237752

[ref8] Dorazi R. Gotz D. Munro S. Bernander R. White M. F. (2007). Equal rates of repair of DNA photoproducts in transcribed and non-transcribed strands in *Sulfolobus solfataricus*. Mol. Microbiol. 63, 521–529. doi: 10.1111/j.1365-2958.2006.05516.x, 17163966

[ref9] Dulermo R. (2025). Archaeal DNA replication initiation: bridging LUCA'S legacy and modern mechanisms. Front. Microbiol. 16:1561973. doi: 10.3389/fmicb.2025.1561973, 40046299 PMC11880632

[ref10] Fariselli P. Taccioli C. Pagani L. Maritan A. (2021). DNA sequence symmetries from randomness: the origin of the Chargaff's second parity rule. Brief. Bioinform. 22, 2172–2181. doi: 10.1093/bib/bbaa041, 32266404 PMC7986665

[ref11] Flynn K. M. Vohr S. H. Hatcher P. J. Cooper V. S. (2010). Evolutionary rates and gene dispensability associate with replication timing in the archaeon Sulfolobus islandicus. Genome Biol. Evol. 2, 859–869. doi: 10.1093/gbe/evq068, 20978102 PMC3000693

[ref12] Gehring A. M. Astling D. P. Matsumi R. Burkhart B. W. Kelman Z. Reeve J. N. . (2017). Genome replication in *Thermococcus kodakarensis* independent of Cdc6 and an origin of replication. Front. Microbiol. 8:2084. doi: 10.3389/fmicb.2017.02084, 29163389 PMC5663688

[ref13] Grigoriev A. (1998). Analyzing genomes with cumulative skew diagrams. Nucleic Acids Res. 26, 2286–2290. doi: 10.1093/nar/26.10.2286, 9580676 PMC147580

[ref14] Grigoriev A. (1999). Strand-specific compositional asymmetries in double-stranded DNA viruses. Virus Res. 60, 1–19. doi: 10.1016/S0168-1702(98)00139-7, 10225270

[ref15] Grigoriev A. (2004). Mutational patterns correlate with genome organization in SARS and other coronaviruses. Trends Genet. 20, 131–135. doi: 10.1016/j.tig.2004.01.009, 15049309 PMC7127256

[ref16] Hawkins M. Malla S. Blythe M. J. Nieduszynski C. A. Allers T. (2013). Accelerated growth in the absence of DNA replication origins. Nature 503, 544–547. doi: 10.1038/nature12650, 24185008 PMC3843117

[ref17] Hogrel G. Lu Y. Laurent S. Henry E. Etienne C. Phung D. K. . (2018). Physical and functional interplay between PCNA DNA clamp and Mre11-Rad50 complex from the archaeon *Pyrococcus furiosus*. Nucleic Acids Res. 46, 5651–5663. doi: 10.1093/nar/gky322, 29741662 PMC6009593

[ref18] Hogrel G. Lu Y. Alexandre N. Bossé A. Dulermo R. Ishino S. . (2020). Role of RadA and DNA polymerases in recombination-associated DNA synthesis in hyperthermophilic Archaea. Biomolecules 10, 1–17. doi: 10.3390/biom10071045, 32674430 PMC7407445

[ref19] Hubert B. (2022). SkewDB, a comprehensive database of GC and 10 other skews for over 30,000 chromosomes and plasmids. Sci Data 9:92. doi: 10.1038/s41597-022-01179-8, 35318332 PMC8941118

[ref20] Ishino S. Skouloubris S. Kudo H. l'Hermitte-Stead C. Es-Sadik A. Lambry J. C. . (2018). Activation of the mismatch-specific endonuclease EndoMS/NucS by the replication clamp is required for high fidelity DNA replication. Nucleic Acids Res. 46, 6206–6217. doi: 10.1093/nar/gky460, 29846672 PMC6159515

[ref21] Karlin S. (1999). Bacterial DNA strand compositional asymmetry. Trends Microbiol. 7, 305–308. doi: 10.1016/S0966-842X(99)01541-3, 10431198

[ref22] Kono N. Tomita M. Arakawa K. (2018). Accelerated laboratory evolution reveals the influence of replication on the GC skew in *Escherichia coli*. Genome Biol. Evol. 10, 3110–3117. doi: 10.1093/gbe/evy237, 30371772 PMC6263442

[ref23] Koonin E. V. Krupovic M. Ishino S. Ishino Y. (2020). The replication machinery of LUCA: common origin of DNA replication and transcription. BMC Biol. 18:61. doi: 10.1186/s12915-020-00800-9, 32517760 PMC7281927

[ref24] Kopejtka K. Lin Y. Jakubovicova M. . (2019). Clustered core- and pan-genome content on Rhodobacteraceae chromosomes. Genome Biol. Evol. 11, 2208–2217. doi: 10.1093/gbe/evz138 31273387 PMC6699656

[ref25] Lobry J. R. (1996). Asymmetric substitution patterns in the two DNA strands of bacteria. Mol. Biol. Evol. 13, 660–665. doi: 10.1093/oxfordjournals.molbev.a025626, 8676740

[ref26] Lopez P. Philippe H. Myllykallio H. Forterre P. (1999). Identification of putative chromosomal origins of replication in Archaea. Mol. Microbiol. 32, 883–886. doi: 10.1046/j.1365-2958.1999.01370.x, 10361290

[ref27] Lu J. Salzberg S. L. (2020). SkewIT: the skew index test for large-scale GC skew analysis of bacterial genomes. PLoS Comput. Biol. 16:e1008439. doi: 10.1371/journal.pcbi.1008439, 33275607 PMC7717575

[ref28] Mackwan R. R. Carver G. T. Drake J. W. Grogan D. W. (2007). An unusual pattern of spontaneous mutations recovered in the halophilic archaeon *Haloferax volcanii*. Genetics 176, 697–702. doi: 10.1534/genetics.106.069666, 17194771 PMC1893060

[ref29] Matsunaga F. Norais C. Forterre P. Myllykallio H. (2003). Identification of short 'eukaryotic' Okazaki fragments synthesized from a prokaryotic replication origin. EMBO Rep. 4, 154–158. doi: 10.1038/sj.embor.embor732, 12612604 PMC1315830

[ref30] Mc Teer L. Moalic Y. Cueff-Gauchard V. Catchpole R. Hogrel G. Lu Y. . (2024). Cooperation between two modes for DNA replication initiation in the archaeon *Thermococcus barophilus*. MBio 15:e0320023. doi: 10.1128/mbio.03200-23, 38421162 PMC11005403

[ref31] Moalic Y. Reveil M. Kundnani D. L. Balachander S. Yang T. Gombolay A. . (2025). Genome-wide ribonucleotide detection in Archaea. Nucleic Acids Res. 53:gkaf1231. doi: 10.1093/nar/gkaf1231, 41273176 PMC12639248

[ref32] Mottez C. Puech R. Flament D. . (2023). Structuring effects of archaeal replication origins. bioRxiv: 2023.11.15.567178. doi: 10.1101/2023.11.15.567178

[ref33] Myllykallio H. Lopez P. Lopez-Garcia P. . (2000). Bacterial mode of replication with eukaryotic-like machinery in a hyperthermophilic archaeon. Science 288, 2212–2215. doi: 10.1126/science.288.5474.2212 10864870

[ref34] Nakae S. Hijikata A. Tsuji T. Yonezawa K. Kouyama K. I. Mayanagi K. . (2016). Structure of the EndoMS-DNA complex as mismatch restriction endonuclease. Structure 24, 1960–1971. doi: 10.1016/j.str.2016.09.005, 27773688

[ref35] Norais C. Hawkins M. Hartman A. L. Eisen J. A. Myllykallio H. Allers T. (2007). Genetic and physical mapping of DNA replication origins in *Haloferax volcanii*. PLoS Genet. 3:e77. doi: 10.1371/journal.pgen.0030077, 17511521 PMC1868953

[ref36] O’Donnell M. Langston L. Stillman B. (2013). Principles and concepts of DNA replication in bacteria, archaea, and eukarya. Cold Spring Harb. Perspect. Biol. 5:a010108. doi: 10.1101/cshperspect.a010108, 23818497 PMC3685895

[ref37] Ohbayashi R. Hirooka S. Onuma R. Kanesaki Y. Hirose Y. Kobayashi Y. . (2020). Evolutionary changes in DnaA-dependent chromosomal replication in Cyanobacteria. Front. Microbiol. 11:786. doi: 10.3389/fmicb.2020.00786, 32411117 PMC7198777

[ref38] Ren B. Kuhn J. Meslet-Cladiere L. . (2009). Structure and function of a novel endonuclease acting on branched DNA substrates. EMBO J. 28:19609302, 2479–2489. doi: 10.1038/emboj.2009.192 PMC2735178 19609302

[ref39] Robinson N. P. Bell S. D. (2007). Extrachromosomal element capture and the evolution of multiple replication origins in archaeal chromosomes. Proc. Natl. Acad. Sci. USA 104, 5806–5811. doi: 10.1073/pnas.0700206104, 17392430 PMC1851573

[ref40] Rocha E. P. Touchon M. Feil E. J. (2006). Similar compositional biases are caused by very different mutational effects. Genome Res. 16, 1537–1547. doi: 10.1101/gr.5525106, 17068325 PMC1665637

[ref41] Rudner R. Karkas J. D. Chargaff E. (1968). Separation of *B. subtilis* DNA into complementary strands. 3. Direct analysis. Proc. Natl. Acad. Sci. USA 60, 921–922. doi: 10.1073/pnas.60.3.921, 4970114 PMC225140

[ref42] Saier M. H. Jr. (2019). Understanding the genetic code. J. Bacteriol. 201:e00091-19. doi: 10.1128/JB.00091-19 31010904 PMC6620406

[ref43] Soppa J. (2022). Non-equivalent genomes in polyploid prokaryotes. Nat. Microbiol. 7, 186–188. doi: 10.1038/s41564-021-01034-3 34949829

[ref44] Tomasch J. Kopejtka K. Shivaramu S. . (2024). On the evolution of chromosomal regions with high gene strand bias in bacteria. MBio 15:e0060224. doi: 10.1128/mbio.00602-24 38752745 PMC11237797

[ref45] White M. F. Allers T. (2018). DNA repair in the archaea-an emerging picture. FEMS Microbiol. Rev. 42, 514–526. doi: 10.1093/femsre/fuy020 29741625

[ref46] Wu Z. Liu J. Yang H. Xiang H. (2014). DNA replication origins in archaea. Front. Microbiol. 5:179. doi: 10.3389/fmicb.2014.00179, 24808892 PMC4010727

[ref47] Yang H. Wu Z. Liu J. Liu X. Wang L. Cai S. . (2015). Activation of a dormant replication origin is essential for *Haloferax mediterranei* lacking the primary origins. Nat. Commun. 6:8321. doi: 10.1038/ncomms9321, 26374389 PMC4595724

