# Supplementary material for: On the origins and variation of nucleotide skews of archaeal genomes
Source: Front Microbiol. 2026 Mar 5;17:1727296. doi: 10.3389/fmicb.2026.1727296 (PMC12999951; doi:10.3389/fmicb.2026.1727296)
Supplement: Supplementary file 1 [file Data_Sheet_1.docx]

**Supplementary materials**

**Supplementary Table 1: Statistical data on the genomes included in the SkewDB.**

| **Category** | **Description** | **Value** |
| --- | --- | --- |
| **General** | Number of sequences | 53837 |
|  | Archaea sequences count | 874 |
|  | Bacteria sequences count | 52963 |
| **Archaeal chromosomal sequences** | Count | 601 |
|  | Mean size | 2639718.84359401 |
|  | Standard deviation of size | 902302.2066048206 |
|  | Maximum size | 5751491 |
|  | Minimum size | 307703 |
| **Archaeal plasmid sequences** | Count | 273 |
|  | Mean size | 353565.73992673995 |
|  | Standard deviation of size | 184830.95033409947 |
|  | Maximum size | 1617346 |
|  | Minimum size | 150468 |
| **Bacterial chromosomal sequences** | Count | 43682 |
|  | Mean size | 3899613.22693558 |
|  | Standard deviation of size | 1779815.1422531186 |
|  | Maximum size | 14782124 |
|  | Minimum size | 150141 |
| **Bacterial plasmid sequences** | Count | 9281 |
|  | Mean size | 340600.75207412994 |
|  | Standard deviation of size | 346184.6336115427 |
|  | Maximum size | 5519592 |
|  | Minimum size | 150008 |

**Supplementary Figure 1:**

**Figure legend:** A violin plot for the distribution of Skew Index (SkewI) values for different taxonomic levels calculated using the sequences that passed the quality control.
